# Applying machine classifiers to update searches: Analysis from two case studies

**DOI:** 10.1002/jrsm.1537

**Published:** 2021-11-25

**Authors:** Claire Stansfield, Gillian Stokes, James Thomas

**Affiliations:** ^1^ EPPI‐Centre, UCL Social Research Institute University College London London UK

**Keywords:** information retrieval, supervised machine learning, systematic reviews as topic, update search

## Abstract

Manual screening of citation records could be reduced by using machine classifiers to remove records of very low relevance. This seems particularly feasible for update searches, where a machine classifier can be trained from past screening decisions. However, feasibility is unclear for broad topics. We evaluate the performance and implementation of machine classifiers for update searches of public health research using two case studies. The first study evaluates the impact of using different sets of training data on classifier performance, comparing recall and screening reduction with a manual screening ‘gold standard’. The second study uses screening decisions from a review to train a classifier that is applied to rank the update search results. A stopping threshold was applied in the absence of a gold standard. Time spent screening titles and abstracts of different relevancy‐ranked records was measured. Results: Study one: Classifier performance varies according to the training data used; all custom‐built classifiers had a recall above 93% at the same threshold, achieving screening reductions between 41% and 74%. Study two: applying a classifier provided a solution for tackling a large volume of search results from the update search, and screening volume was reduced by 61%. A tentative estimate indicates over 25 h screening time was saved. In conclusion, custom‐built machine classifiers are feasible for reducing screening workload from update searches across a range of public health interventions, with some limitation on recall. Key considerations include selecting a training dataset, agreeing stopping thresholds and processes to ensure smooth workflows.

**What is already known**

Using a machine classifier for an update search is feasible in some contexts.

**What is new**

Describes and evaluates the utility and implementation of classifiers to update searches for a broad topic area of public health intervention research.

**Potential impact**

The study informs the implementation of applying machine classifiers to update searches for systematic reviews and living systematic reviews. Key considerations include selecting an appropriate classifier, agreeing stopping thresholds and using information management processes to ensure smooth workflows.

## 1 INTRODUCTION

Identifying research for inclusion into systematic reviews and research registers with a wide public health intervention focus can involve sensitive searching to retrieve a high recall of a wide range of literature.^1^, ^2^ This also entails screening thousands of irrelevant citation records, which takes considerable time if undertaken manually. Machine learning is a possible way of reducing workload as it can be used to rank the records by relevance so that those of very low relevance are not manually screened.^3^ Machine learning requires data from which to ‘learn’, so one promising use scenario is in the case of update searches, where previous screening decisions applied in the original review are available. This is particularly appealing for research topics where systematic literature searches yield many irrelevant records.

The model of identifying studies for systematic reviews is based upon retrieving citation records identified from a literature search followed by screening their titles and abstracts for relevance against pre‐defined eligibility criteria. Those that meet the criteria are screened again based on the full‐text publication. In a traditional approach, humans screen all the records retrieved from a systematic search, within the time and resource available. The literature search strategy is designed in a way to achieve a manageable volume of records from the search results for human screening. Approaches to reduce the volume of records for screening include adjusting search terms and search syntax, the number of resources searched, or other parameters such as date limits. This approach is particularly challenging where a search yields relatively high volumes of irrelevant citation records, and there is no way to modify the search strategy without reducing recall. Machine learning has the potential to help here by ranking the search results returned according to relevance, with those deemed highly likely to be irrelevant not requiring human assessment. In both systematic searching and machine learning, there is uncertainty around any research that is missed, and each approach requires judgements on implementation.

In this paper, we present two case studies where custom‐built machine classifiers (generated from project‐specific datasets) were applied to update searches that aimed to identify a broad range of public health intervention research. We begin by briefly describing the custom‐built classifiers and their application to update searches followed by the case studies. The first case study compares recall and screening reduction obtained when using different classifiers against gold standards of manual screening. The second study reflects on applying a classifier for a systematic review update search, and applying a stopping threshold to cease manual screening in the absence of a gold standard.

## 2 BACKGROUND: DEVELOPING AND APPLYING MACHINE CLASSIFIERS FOR UPDATE SEARCHES

Machine classifiers are a type of ‘supervised’ machine learning which depend upon training data upon which to ‘learn’. In our case studies, they are ‘trained’ using screening data generated from human screeners. A sufficiently large volume of screening data is needed to achieve a good classifier performance. Some machine classifiers have been developed from large sets of data to recognise certain characteristics such as study design. For example, a machine classifier for identifying randomised controlled trials (RCTs) has been developed from over 280,000 health‐related citation records that were classified by humans in the Cochrane Crowd.^4^ By applying a threshold to remove records that are highly unlikely to be describing RCTs, at least 40% of records were able to be discarded from a typical search without undertaking manual screening, while achieving over 99% recall.^4^ However, as this classifier can only identify RCTs in health research, it cannot be used to identify other study designs^5^ or achieve similar recall of RCTs in other domains (e.g., we observed this from retrospectively applying it to RCTs in a systematic review of education research, where performance was unacceptably low).

Unfortunately, large quantities of high‐quality training data are not available for many use scenarios. Machine learning can still be useful though, and in the case of update searches, can utilise the screening data generated from the original search(es). We describe these as ‘custom‐built’ classifiers, as they are trained on use‐specific data, and are not intended to generalise beyond their specific project. While they tend not to have the scale of training data that was available in, for example the RCT Classifier above^5^ (and so might not be as accurate), they have the potential to be more tailored for the task in hand; for example, in our use cases, they can cover a wider range of study designs.

When applied to ‘unseen’ records, a machine classifier can output a score for each record indicating how relevant it is to the class of interest (e.g., an RCT classifier will rank a record on how likely it is to describe an RCT). The score output is on a continuous scale rather than a binary decision of relevance. This score can then be converted into a binary decision by applying a ‘threshold’, below which the citation records will not be screened. Applying a threshold is a key challenge when using any machine classifier in order to maximise recall and reduce manual workload. Decisions might be informed by user‐assessment, heuristics or statistical approaches.^6^

Using a machine classifier for an update search is feasible where the scope of the update search is unchanged from the original search, and where there is no shift in terminology used in the research field, and if the training data seems ‘large enough’.^7^ This approach may be less feasible where the data from the original search have a different scope to the update, such as in cases where there is a ‘concept drift’ from changes in the research team, the searches, and aspects of the review question.^3^, ^8^There are a variety of different methods for machine classification^3^, ^9^ for example, support‐vector machine (SVM), Naïve Bayes, neural networks and ensemble methods. Convolutional neural networks (CNNs) have been shown to have a marginally better performance than SVM for classifying RCTs though this also depends on the model parameters chosen, the data available, and the threshold used to determine likely relevance.^4^ It is not the intention in this paper to evaluate these different methods though, and their performance can depend on context. Wallace et al.^7^ suggest using a ten‐fold cross validation analysis to assess the performance of a classifier model before applying it to an update search. They suggest the training data are split so that 90% trains a classifier that is then tested on the remaining 10%, with the process repeated 10 times. Performance is estimated as the average of results across the 10 tests.

Shekelle et al.^10^ used machine classifiers for three systematic review updates compared with a manual screening approach. The authors trained the classifier based on inclusion decisions in the final report, and found that two studies were missed in three reviews, and that the volume of citation records for screening was reduced by between 67% and 83%. They suggest that the two studies missed did not alter the review conclusions or the strength of the evidence. Wallace et al.^7^ estimated a screening reduction of between 70% and 90% for four update searches, and one study was missed. These studies show that machine learning is a promising approach especially where the volume of records needing to be screened might otherwise be prohibitive. Large reductions in screening are inevitably related to the precision of the search results in individual cases. As systematic review searches within health research have an estimated precision of 3% at full‐text inclusion,^11^ there is an opportunity for time savings from not screening irrelevant citation records in this field. For public health reviews the precision of searches may be lower, due to the language used and the comparatively lower use of technical jargon. For example, one of the case studies presented in this paper is a systematic map of public health interventions by community pharmacies.^12^ The precision of the original search was 1%, based upon 21,329 records screened to locate 255 relevant papers. The interventions are each are described by a wide range of terminology (i.e., they are described by many different words which may not be distinctive to a particular context), and the low precision partly relates to the broad scope of the research considered relevant, the diffuse terminology used to describe it, and its broad classification within information systems.

Applying machine classifiers to update searches on public health interventions is appealing, because of the large numbers of citation records retrieved, though there are few available evaluations so their performance on diffuse topics is uncertain. Questions on their utility include determining an optimal quantity of training data, assessing recall performance, and the workload saved within a given stopping threshold. Questions on implementation include practical considerations in applying the tool and reporting a transparent workflow of study identification.

In the remainder of this paper, we present two case studies that utilise machine classifiers to identify interventions in public health that can inform the above research gaps. The case studies have specific and different purposes. The first case study compares the utility of custom‐built classifiers and the RCT classifier developed from the Cochrane Crowd dataset for identifying controlled trials in public health. It uses manually‐screened citation records as gold standards. The second case study considers both the utility and implementation of using machine classifiers in an update search in the absence of a gold standard and where a threshold for stopping manual screening was developed and applied. We present each study separately, followed by a combined discussion and conclusion.

## 3 CASE STUDY 1: COMPARING PERFORMANCE OF CLASSIFIERS

### 3.1 Background

The Trials Register of Promoting Health Interventions (TRoPHI)^13^ contains citations describing randomised and non‐RCTs of health promotion and public health. Since 2004, the register has been populated from routine searches and from research obtained while conducting systematic reviews. Its purpose is to facilitate the gathering of evidence in health promotion research. As of December 2020, the database contains over 14,000 citation records that meet the register's eligibility criteria. The eligibility criteria are applied to the title and abstract citation record by one human screener. Records that describe health promotion effectiveness reviews are assessed for inclusion in a separate database, Database of Promoting Health Effectiveness Reviews.^14^

### 3.2 Aim

To compare the performance of machine classifiers developed from different training sets in terms of highest recall and highest screening reduction. The results inform which classifier to use for update searches for the TRoPHI database of public health interventions.

### 3.3 Methods

This case study compares the performance of four classifiers: The RCT classifier described above that was built from data generated by Cochrane Crowd; and three custom‐built classifiers that were built from data previously generated within the TRoPHI register. For the three custom‐built classifiers, training and test datasets of human‐coded citation records screened using the TRoPHI register eligibility criteria were used as a gold standard. These records (*n* = 19,759) were from searches undertaken between January 2012 and June 2013, and had been manually assigned to one of multiple options relating to exclude and include (Table 1). The three classifiers were trained from the same dataset of records, but differed in terms of which parameters (screening decisions) informed the training for inclusion as follows: Custom 1: Meets topic and any study design; Custom 2: Meets topic and study design is either a controlled trial or intervention effectiveness review; Custom 3 meets topic and is a controlled trial (see Table 2, column 2). We refer to the three custom‐built classifiers as Custom 1, Custom 2, and Custom 3, respectively.

**TABLE 1:** Description of the training and test sets

Exclusion (Ex) and inclusion criteria for TRoPHI	Training set (*n* = 19,759)^a^	Test set A (*n* = 9,368)	Test set B (*n* = 7,185)	Test set C (*n* = 5,812)
Description	Searches between January 2012 and June 2013	Searches July 2013–March 2015	Searches during 2010, publication date 2009–2010	Searches during 2008, publication date 2007–2008
Ex1: Focus is not on health promotion or public health	16, 536	8502	6796	5351
Ex2: Study is not a prospective evaluation of an intervention	1799			
Ex3: Study has no control or comparison group				
Ex4: Item is a review (consider for Database of Promoting Health Interventions)	312			
Include 1: non‐randomised controlled trial (non‐RCT)	220	213	103	96
Include 2: Randomised controlled trial (RCT) (this includes a true randomised method, or quasi‐randomisation such as alternate allocation)	892	653	286	365

^a^ Classifier was trained on 20,050 references, numbers adjusted following additional duplicate‐removal.

**TABLE 2:** Performance of the classifiers on test set A (*n* = 9368), B (*n* = 7,85) and C (*n* = 5812)

Classifier	Training criteria^a^	Set	RCTs		Non‐RCTs		Screening reduction %
			Precision %	Recall %	Precision %	Recall %	
RCT classifier	Include RCT in any human health domain	A	12.3	99.7	3.4	85.9	43.3
		B	8.1	99.7	2.4	83.5	50.9
		C	11.1	99.2	2.6	87.5	43.6
Custom 1	Include any studies that are in the health promotion domain (all studies without Ex1 code)	A	11.7	99.1	3.8	99.5	40.9
		B	7.8	100%	2.8	100%	49.3
		C	13.6	99.5	3.5	96.9	54.2
Custom 2	Include any RCTs, non‐RCTs or reviews in the health promotion domain (all studies without Ex1, Ex2 or Ex3 codes)	A	16.5	98.8	5.4	98.6	58.4
		B	12.2	99.3	4.3	97.1	67.6
		C	19.6	98.1	4.9	93.8	68.6
Custom 3	Include any studies that are RCTs or non‐RCTs in the health promotion domain (all studies without Ex1, Ex2, Ex3 or Ex4 codes)	A	19.7	98.0	6.3	96.2	65.4
		B	14.6	99.0	5.1	97.1	72.9
		C	23.2	97.8	5.8	92.7	73.5

^a^ Ex1, Ex2, Ex3, Ex4 are described in Table 1.

These were built within the machine classifier function available within EPPI‐Reviewer 4.^15^ This classifier utilises the popular ‘sci‐kit‐learn’ machine learning library and is written in python and deployed on the Azure Machine Learning platform. The decisions made in text preparation can often have a bigger impact on classifier performance than the selection of any particular algorithm. Different options for text pre‐processing were evaluated when it was initially developed, and it was found that a “bag‐of‐words” approach using tri‐grams *without* word stemming provided the most consistently high and generalisable performance. Stop words listed in the PubMed stop‐word list are removed. In our use case, this permits the classifier to recognise “randomized controlled trial” as a specific term, something which would be lost if uni‐or bi‐grams were chosen. The lack of stemming is helpful too, in that it enables the model to be ‘aware’ of the difference between records that describe “randomized controlled trials” and a single “randomised controlled trial”—a distinction that would be lost if words were stemmed. This of course helps the model to distinguish between discussions of multiple trials—for example in systematic reviews—and presentations of the results of a single trial. The approach we used in these case studies was to use the ‘SGDClassifier’, which can be used to implement logistic regression and SVM models. In our case, we used the logistic regression model and multipled the output probabilities by 100 to give the relevance scores presented in this paper.^16^

The above four classifiers were evaluated by applying to three separate datasets of records, which were generated from searches carried out during 2008, 2010 and between July 2013 and 2015. These datasets are referred to as ‘A', ‘B' and ‘C'. Table 1 describes the datasets and shows the distribution of the exclude and include codes within the training and test sets. Duplicate‐checking was undertaken between each dataset to avoid overlap of samples. The classifiers generated a relevance ranking score for each citation in the test set of between 0 and 99, where 99 is highly relevant. A threshold was applied to exclude those that ranked as having ‘very low relevance’, between 0 and 10. Precision and recall of RCTs and non‐RCTs (non‐RCTs) in the test set and screening reduction was calculated as defined in Box 1.

**BOX 1:**
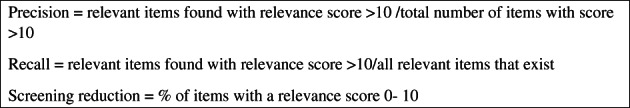
Definitions of the performance parameters

### 3.4 Results

Table 2 shows the performance of the classifiers for the controlled trials on the test sets. The RCT classifier achieved at least 99% recall across all samples, Custom 1 had marginally better recall of RCTs for two of the three test sets. All classifiers achieved at least 98% recall of RCTs. For recall of non‐RCTs, the custom‐built classifiers performed better than the RCT classifier, with at least 93% recall. This is as expected, as the RCT classifier was not designed for this purpose.

Screening reduction across the classifiers ranged from 41% to 65% for the test sample A, and from 44% to 73% across test samples B and C. For the custom‐built classifiers there is a trade‐off between higher screening reductions and lower recall, and this trend is more marked for non‐RCTs. Furthermore, there is variation between the results of the test sets for each classifier, particularly in identifying non‐RCTs. It is possible this is owing to the wide variation in terminology of non‐RCTs combined with the relatively small number of studies that are non‐RCTs in the training and test sets. The classifiers were trained on 220 non‐RCTs, compared with 892 RCTs. The RCT classifier had a lower precision of RCTs, and lower screening reduction compared with Custom 2 and 3, indicating the influence of subject domain in contributing to performance.

Since this analysis, the Custom 2 classifier has routinely been applied to update searches of the trials register. While the recall is less than Custom 1, it achieves a higher screening reduction. It may be possible to boost performance by training a new classifier using citation records with screening data generated since this study was undertaken.

## 4 CASE STUDY 2: IMPLEMENTING A MACHINE‐CLASSIFIER WITHIN A SYSTEMATIC REVIEW WORKFLOW

### 4.1 Background

An 18‐month update search was undertaken for a systematic map of public health service provision by community pharmacies, prior to publication.^12^ The map summarises research studies that examine the effectiveness and appropriateness of community pharmacies in providing public health services to local populations. An update search was considered necessary as we anticipated a growth of research in this area. The update search yielded 23,208 additional records after an initial removal of duplicate records that had been identified in the original review. This search yield was higher than obtained for the original search (21,329 records). The search yield contained many irrelevant items, though there was no clear way to reduce the number of records it generated to lie within the resources available for manual screening. One option was to re‐construct and test the entire search to try to reduce the yield, and another option was to train and apply a machine classifier in order to reduce manual screening. Both options required decisions on setting limits, either setting limits around the searches or setting limits on when to cease screening. We considered the latter approach as the most feasible.

Locating literature on community pharmacy public health provision is challenging. The literature search was based on two concepts: ‘Community pharmacy’ and ‘public health’, and each are described by diffuse terminology. For example, retail pharmacies located on shopping streets or in supermarkets may not be labelled as a community pharmacy, and they provide a variety of services within the remit of public health. Public health in this context concerns many interventions to promote a healthy lifestyle, including services for diabetes and cardiovascular health, immunisations, sexual health, substance misuse and antimicrobial resistance awareness, among others. These services include capacity‐building interventions, such as providing health champions to engage with service providers and local communities, and individually‐delivered interventions such as health education to promote a healthy lifestyle or reduce specific health conditions. Prior to the update, the original database search was checked against relevant studies that were identified from outside the database search to assess why they were not located from the database searches. This informed an expansion of the update search by searching additional sources (Emerging Sources Citation Index, International Pharmaceutical Abstracts and additional websites) and removing certain database limits that were originally applied in some of the health database searches in order to reduce the volume of results. Therefore, the search was updated with the entire timeframe of the review, from 2000 to 2017. The scope of the review, the search concepts, search terms and syntax remained unchanged from the original search.

### 4.2 Aims

(1) To describe the issues encountered, decisions and results from applying machine classifiers to facilitate prioritised relevance screening against the eligibility criteria for the map, and to assess the screening saved from not screening search results with a low relevancy score. (2) To estimate the time saved from not screening citation records with a low relevancy score. (3) To consider the implementation of the process within the systematic review workflow.

### 4.3 Methods

#### 4.3.1 Selecting a classifier

Two custom‐built classifiers were developed using the machine classifier function within EPPI‐Reviewer 4 (with the same characteristics as described in the previous methods section).^15^ The classifiers were trained on the screening decisions from the original map and applied to the search results of the database update searches. The first classifier was trained on the screening decisions of citation records at the title and abstract stage, using 894 titles and abstracts as the basis of an include decision, and 20,435 title and abstracts as excludes. A second classifier was trained on the title and abstracts records from the full‐text includes and relevant systematic reviews (*n* = 261) as the basis of an include decision, and the remaining records as the basis of an exclude decision (*n* = 21,068). The EPPI‐Reviewer 4 interface provides a bar chart showing the distribution of the citation records across the relevance scores, and this was used to indicate the suitability of each classifier (presented in the results section). At this point, the first classifier was determined as not suitable, and the second classifier was applied to the update search.

The second classifier was retrospectively tested using a stratified five‐fold cross‐validation analysis using the gold standard data, by training on a 90% sample of the original training dataset and testing on the remaining 10% of the training dataset to check the ranking scores of the known relevant records. The sets were generated from random samples of the 261 includes and 21,068 excludes in the training data, repeated to obtain five sets of training and test sets with recall being the statistic evaluated.

#### 4.3.2 Applying the classifier to the update search

Out of the 23,208 citation records from the database searches, 21,420 contained titles and abstracts and were ranked by relevance using the second classifier. A bar chart showing the distribution of records across the relevance scores informed the development of an algorithm to set a threshold below which screening would cease. We intended to cease screening after a predetermined interval where no further relevant records were identified. However, if this interval was not achieved, screening would cease after a specified number of records in agreement with the review team. The algorithm comprised of the following three rules: (1) Do not screen records with a score of 10 or less; (2) Manually screen records with a score of 20 or more; (3) Manually screen records with a score of 11–19 in batches of 500, starting from those with a score of 19; (4) Screen a further 1000 records after the last include. During the process of manual screening, a modification to rule 4 was imposed: to only screen the batches of 500 until score 13 as the interval of 1000 was not achieved.

#### 4.3.3 Evaluating performance

The performance of the second classifier on the update search results was retrospectively assessed on 21,403 citations. Total screening reduction was calculated, based on the number of citation records that were not screened. The precision of the relevance scores for the relevant records was determined. As all records were not screened it is not possible to calculate recall. We compared the inclusion rate (precision) and publication date of the included studies from relevance‐ranked screening with other records that were screened manually, which were from: the original searches, records that were title‐only (n=1,788)and searching and browsing websites (n=15). Reflection on implementing the process into the review update workflow was undertaken throughout.

#### 4.3.4 Time analysis

We measured the time taken by one reviewer to screen 40 abstracts (eight samples of five abstracts) in a set of references with relevance scores between 13 and 19. This was compared with the time taken to screen 40 abstracts across sets of references with higher relevance scores between 20 and 99 (eight samples of five abstracts across this range). The time taken to screen each abstract was measured in seconds by one reviewer using a digital stopwatch. The stopwatch was started the when the reviewer's eyes first met the screen and stopped when the reviewer reached a decision on the exclude code. There were 14 exclude codes that the reviewer could choose from, or an include code. Exclude codes consisted of study design, publication type or date, country, and specific topic exclusions. The screening was undertaken in conjunction with a ‘show terms’ feature in EPPI‐Reviewer 4 that highlights terms pre‐determined by the user as relevant in green and those that are irrelevant in red (the relevant and irrelevant terms were determined during the original screening, prior to the update search). The reviewer had screened a significant number of studies from the original review and so had a high level of familiarity with the types of abstracts that would be encountered in the screening and the criteria used for each of the exclusion and inclusion codes.

### 4.4 Results

#### 4.4.1 Selecting a classifier

**FIGURE 1:**
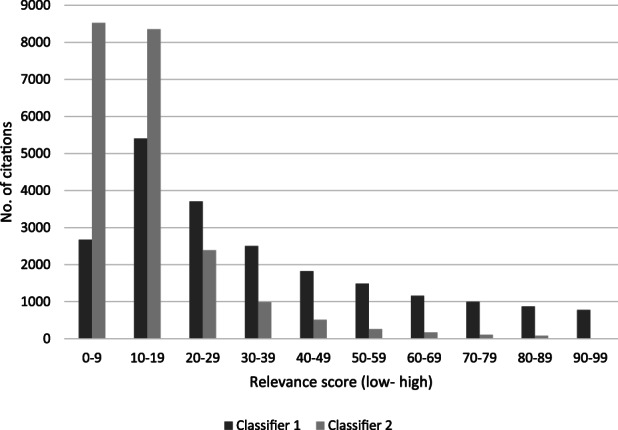
Relevance scores of two classifiers (*n* = 21,404)

Figure 1 shows the bar charts of the two classifiers. The second classifier was considered suitable, as it showed the highest number of citations were within the lower relevance score range, and a marked decrease in the higher relevance rankings from 20 to 99. Classifier 1 did not display this same trend as there were less citations marked as of very low relevance (0–9) than scores of (10–29) and therefore seemed less precise. This was not surprising as the training data for classifier 1 was based on inclusion decisions at title and abstract. When the studies were screened at full‐text a series of additional exclusion criteria were applied, and these stricter criteria informed the training of Classifier 2 (e.g., these criteria included medication management except for antimicrobial resistance, all process evaluations and views studies outside UK settings).

The five‐fold cross‐validation analysis of the second classifier showed an average recall of 99% (range 100%–96%, based on 26 or 27 included studies per set) using a threshold score of above 14: across two tests one included study had a relevance score of 9 and another study scored 14.

#### 4.4.2 Applying the classifier to the update search

Out of the 23,208 citations from the searches, 21,455 were eligible for the machine classifier. This was reduced to 21,403 following further identification of duplicates during manual screening. It was noticed that some citations contained abstracts in the notes field rather than in the abstract field, including 5786 records from one database. These were checked and edited within a citation management tool prior to applying the classifier. All the records to be screened were labelled and distributed between two reviewers for single‐screening. The reviewers had both screened for the original review and had experience in screening from other systematic reviews on public health topics. This labelling was particularly crucial for applying the stopping rules, as records with a score of under 19 were screened in order of relevance ranking in order to inform when to stop screening. The screening was undertaken in batches of 500 at relevance scores of 19 and lower, until those at score 13 and under remained. In the last manually‐screened batch of 500 citations, of which some were score 13, one citation was identified as relevant on title and abstract, but on full‐text retrieval was considered irrelevant. Although the original algorithm was designed to help to inform when to stop manual screening, it was still too inclusive without modification, due to some ambiguous abstracts.

#### 4.4.3 Evaluating performance

**FIGURE 2:**
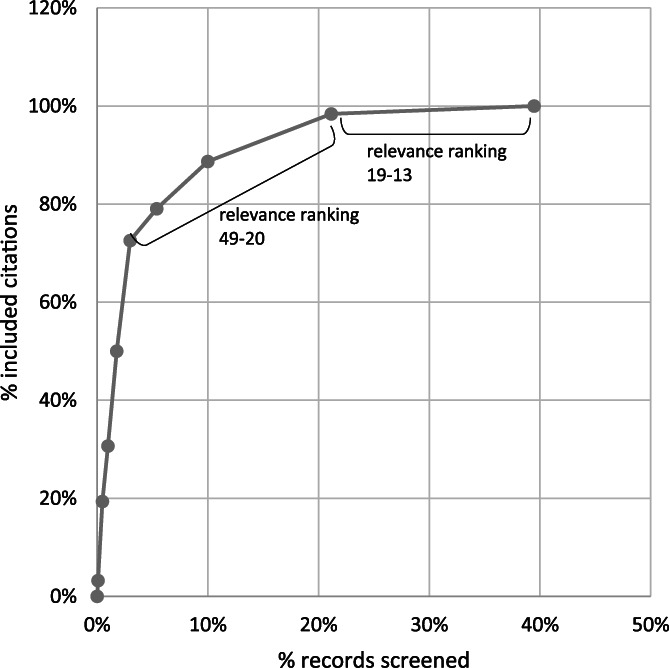
Relevant records per volume screened (*n* = 8,449)

**FIGURE 3:**
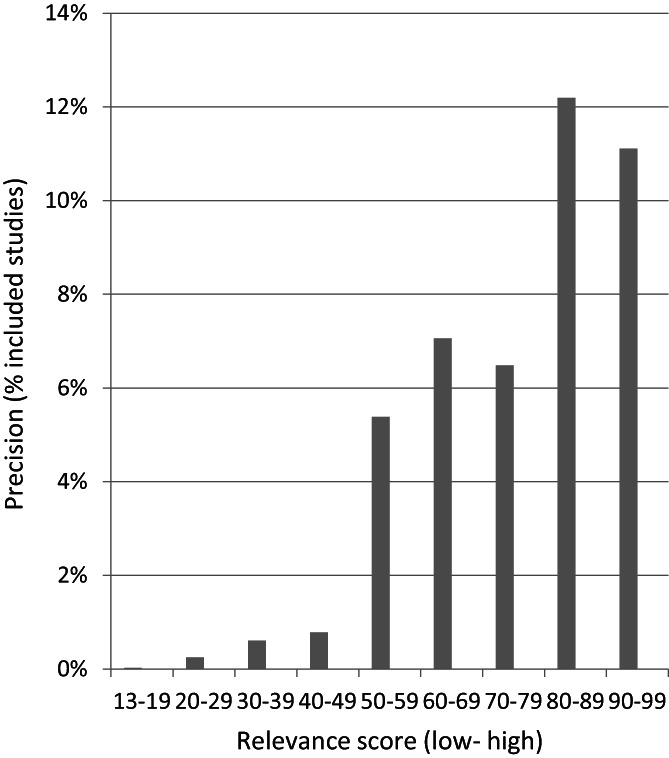
Precision of the 62 relevant records within relevance ranking scores

Out of the 21,403 title and abstract records, 8449 were manually screened, corresponding to a screening reduction of 61%. Of the title and abstract records screened, 62 records, reporting 55 studies, were included in the systematic map. Figure 2 shows the distribution of relevant records per references screened. The machine classifier ranked 61 (98%) of these citations within the first 21% citations for screening, and these had a relevance score of between 20 and 99. The remaining citation that was included in the systematic map had a score of 14. Seventeen references (27%) were identified between the mid‐ranking relevance scores 40–49 to the low‐relevance score of 13. Figure 3 shows the precision of the relevance scores of citations included in the systematic map based on the total citations within each relevance score range. As expected, the higher precision was achieved at higher relevance scores, than at the lower scores from 13 to 49, though these lower scores were clearly important for identifying 27% of references.

The study that had a relevance score of 14 related to compliance with antibiotic therapy.^17^ Antimicrobial resistance is one of the priority areas identified by the funder of the systematic map^12^ and only one study on antibiotic therapy was in the original map. Three studies on antimicrobial resistance were found from the update search, one each from the expedited title and abstract screening.^17^ one from the screen of citations without abstracts^18^ and one from the website searching.^19^

**FIGURE 4:**
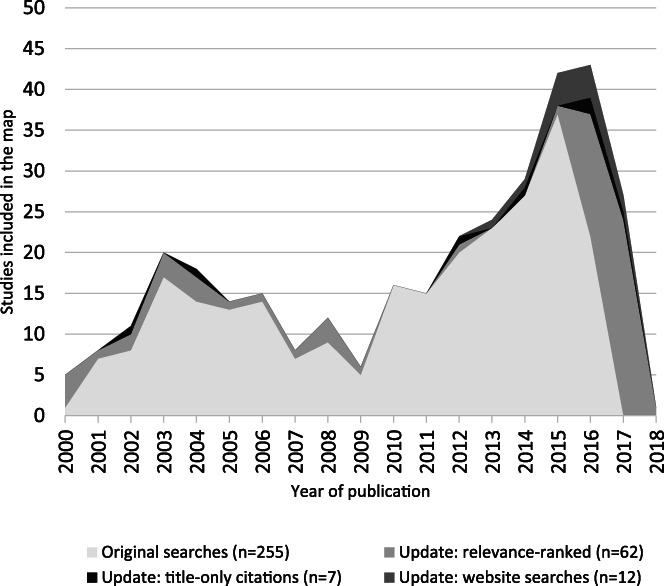
Contribution of all the studies shown by publication year (*N* = 336)

The inclusion rate (precision) of titles and abstracts from the relevance screening process was 0.73%. All citation records that did not contain abstracts were manually screened (*n* = 1,788) to yield a further seven studies for inclusion in the map, which equates to 0.39% precision. A further 12 studies were included in the map that were identified from website searches. Figure 4 shows the contribution of these three groups of citations to the overall set of studies, arranged by publication year.

#### 4.4.4 Time analysis

**FIGURE 5:**
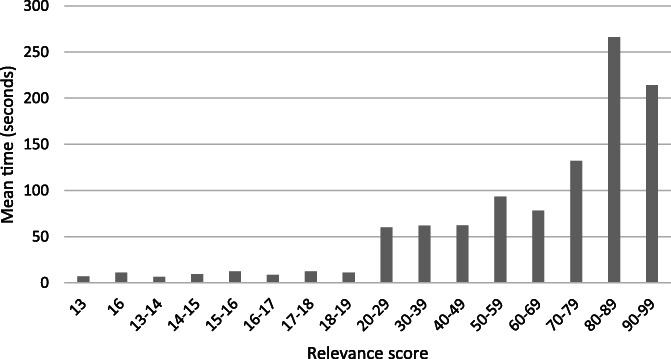
Screening time for records by relevance score (*n* = 5, for each relevance score range)

Table 3 presents the minimum, maximum and mean time taken to screen a single record, based on screening five records per relevance score range. Figure 5 shows the distribution of mean screening time for studies by relevance score. Overall, there is a trend for longer times to screen records with high relevance scores compared with those of lower relevance. 12,954 titles and abstracts were not screened by the reviewers. Based on the mean time of 7 s to screen the records with a low relevance score of 13, this equates to an estimated 25 h of screening time saved.

**TABLE 3:** Time taken to screen citation records at different relevance scores

	Screening time per record based on five per category (seconds)			
Relevance score	Minimum	Maximum	Mean	Total
90–99	138	280	214.2	1071
80–89	198	320	266	1330
70–79	15	303	132.2	661
60–69	30	132	78.4	392
50–59	35	134	93.2	466
40–49	10	222	62	310
30–39	10	140	61.8	309
20–29	8	130	59.8	299
18–19	7	15	11.2	56
17–18	5	17	12.2	61
16–17	5	15	8.4	42
16	3	18	11	55
15–16	4	30	12.2	61
14–15	5	15	9.4	47
13–14	5	8	6.6	33
13	3	15	7	35

### 4.5 Discussion

#### 4.5.1 Developing and testing classifiers

In the first case study, testing the precision and recall provided confidence in selecting a classifier and applying it to update searches, though the variation in performance across the test sets highlight limitations in achieving recall. There are also decisions around how inclusive the training dataset should be. When using screening data generated from human screeners who adopt a hierarchical screening process it may be possible to evaluate and select from a number of inclusion and exclusion criteria, which may have different implications for classifier performance. In case study one, although the Custom 3 classifier was trained to be more specific to the study design of interest than Custom 1 and 2, Custom 3 had lower precision and recall for non‐RCTs and slightly lower recall of RCTs than Custom 2.

In the second case study, post‐hoc testing of the chosen classifier achieved a high recall though could still potentially miss some studies. There will inevitably be some uncertainty in the development and suitability of custom‐built classifiers owing to the relatively small datasets they are based on, and possible variations in the parameters applied. Observing a bar chart showing the distribution of the relevance scores from applying a classifier helps reviewers assess the suitability of the classifier (such as shown in Figure 1). Cross‐validation analyses provide a quantitative indication of recall performance. Applying a series of stopping rules informs performance, though this occurs during the screening process, once the classifier has been selected and applied.

#### 4.5.2 Applying the classifier

Case study one had the benefit of comparison with a gold standard. The variation in recall across datasets between 93% and 98% for non‐RCTs shows there are challenges in identifying non‐RCTs. From our experience it can sometimes be difficult to label a record as non‐RCT from reading an abstract alone, and this was also observed by Hausner et al.^20^ as part of obtaining gold standards for search filter development for this study type (which achieved 92% sensitivity^21^). The recall level from a reduction in screening is sufficient for the purposes of the TRoPHI register, which is to support the efficient identification of controlled trials in health promotion, rather than functioning as a single source.

In the second case study it was important to discuss and justify the process of applying a classifier with the review team and the funder of the review. The alternative option of developing a more specific search strategy was not viable, as there was no clear way to restrict the search strategy without losing relevant records. While constructing a precise search would have provided greater transparency than utilising a machine‐learning approach, it seemed less desirable in reaching the goal of mapping the literature. A more precise search would have reduced the possibility of locating relevant studies from a range of contexts or publications or that offer different findings. Evaluating the original search strategy with the results of the original map helped support decisions around the update search and the approach taken.

There is a conceptual and cultural barrier around not screening studies that are identified from literature searches. Undertaking partial screening creates uncertainty over the number of items to screen for review teams, though this can be estimated to some extent at the outset for update searches. Information management processes are needed to ensure smooth workflow of the items to be screened and to implement a stopping criterion. In case study two, stopping rules needed to be developed and modified within the process to manage the workload of screening, and were also informed from feedback while screening was taking place. The finding of one relevant study with a relevance score of 14 shows the importance of screening to a relatively low threshold for broad topics or where studies are not well represented in the original dataset. With hindsight, testing could have been undertaken on the remaining unscreened references to check if there were relevant references (e.g., using unsupervised clustering, or applying a classifier trained using different parameters). In the future we hope to utilise other approaches to determining stopping thresholds from ongoing research within our research centre. Finally, it is important to note that the scores are a relative concept of relevance, and the reported scores used in this study are not intended to be used as thresholds that can be applied in other situations. This point is supported by Weightman et al.^22^ who used the same classifier function within EPPI‐Reviewer 4 to retrospectively observe performance compared with manual screening for two update searches on social care topics; they found they would have obtained 100% retrieval, if they had only screened to threshold scores of 22 and 43, respectively.

#### 4.5.3 Performance of the classifiers

In both case studies the classifiers enabled a reduction in the number of irrelevant records that needed to be screened. Case study one also shows the influence of subject domain in contributing to performance, and a benefit of custom‐built classifiers over generic classifiers to decrease screening volume. While the workload in screening is inevitably reduced, the time savings are less clear. In case study two we made a modest attempt to indicate the type of time savings that the workload reduction could produce.

#### 4.5.4 Screening time analysis

The Cochrane Handbook suggests a “conservatively estimated reading rate” of 30–60 s for abstracts of health interventions, or approximately 500–1000 over an 8‐hour period.^23^ Przybyła et al.^6^ observed lower screening times at the later stages of a prioritised screening process. We also observed a corresponding relationship between relevance score and screening time; however, our data indicate this is not constant for all citation records (based on a low‐sample size). The time needed to screen abstracts with the highest relevance scores in our study was between 15 to 320 s. This variation reflects our experience that some abstracts can be quickly discarded by reading only a title, and others require more processing time to both read and understand the abstract and to also consider how it matches with the eligibility criteria. There is also the possibility that reviewer experience with the screening improves speed.^6^ With priority screening, the records with lower relevance scores were much quicker to screen, with a range of 3–30 s. An estimate of the time saved by not screening 12,954 citations extrapolates to 25 h based on 7 s per record (the mean screening time of the lowest ranked study for screening just five records). However, we expect this time saving to be greater in practice as it does not consider screening at scale, and the need for rest breaks. Other variables that could influence time needed to screen include: concentration level and screening fatigue, internet and computer speeds, reading speed, abstract length, and overall environment in which screening is undertaken. Undertaking a time analysis on larger samples of records, and with more than one screener, would provide a more accurate estimate of time savings.

## 5 LIMITATIONS

For case study two, the reported precision of the title and abstract screening may be marginally higher than reported. Conference abstracts were included in the update searches though removed early on in the screening process. However, we estimate that without this, precision would still be under 1%. Duplicates are a challenge for any search across multiple resources, and particularly for update searches as the search has been undertaken at more than one timepoint. For case study two, the duplicates were removed at the outset of the process, and when identified during screening. We expect there will be some duplicate records that have been missed, particularly those classed as irrelevant. The time analysis in case study two is based on a low sample size.

## 6 CONCLUSIONS

Both case studies show that custom‐built classifiers can achieve considerable reductions of screening for update searches in broad public health intervention topics. They are particularly useful where a yield of search results is difficult to reduce using conventional methods. Case study one demonstrates there is a domain influence in applying classifiers. A custom‐built classifier may achieve higher screening reductions towards a specific domain than a generic classifier derived from larger datasets covering a broader domain. However, achieving high recall and high‐screening‐reduction appear to be limited by the quality of the training data. Questions on the utility of classifiers for update searches include: determining an optimal quantity of training data, assessing recall performance, and assessing workload saved within a stopping threshold. Owing to the customised nature of such classifiers, answers to these questions may vary across different cases. Case study two shows how this can be understood and implemented within the context of updating a systematic map. Our findings suggest that there is potential to significantly reduce time spent screening by applying custom‐built machine classifiers and excluding studies of low relevance using appropriate stopping rules. Key considerations include selecting an appropriate the classifier, agreeing stopping rules and using appropriate information management to ensure smooth workflow.

## CONFLICT OF INTEREST

The authors declare that they have no competing interests.

## AUTHORS' CONTRIBUTIONS

Stansfield C developed and analysed both case studies and drafted the manuscript. Stokes G designed the time analysis component of case study two and contributed to the analysis and interpretation of this case study. Thomas J contributed to the design, analysis and interpretation of the studies. All authors drafted and approved the final manuscript. Thomas J oversees the development of EPPI‐Reviewer, including integration of the machine‐learning tools.

## Data Availability

The datasets used and/or analysed during the current study are available from the corresponding author on reasonable request. The machine classifier source code is published here: https://github.com/EPPI‐Centre/CochraneCOVID19Classifier (this is for the Cochrane COVID19 classifier, but thesame algorithm is used in EPPI‐Reviewer).
